# Bumped kinase inhibitor 1369 is effective against *Cystoisospora suis in vivo* and *in vitro*

**DOI:** 10.1016/j.ijpddr.2019.03.004

**Published:** 2019-04-02

**Authors:** Aruna Shrestha, Kayode K. Ojo, Florian Koston, Bärbel Ruttkowski, Rama S.R. Vidadala, Carlie S. Dorr, Edelmar D. Navaluna, Grant R. Whitman, Kayleigh F. Barrett, Lynn K. Barrett, Matthew A. Hulverson, Ryan Choi, Samantha A. Michaels, Dustin J. Maly, Andrew Hemphill, Wesley C. Van Voorhis, Anja Joachim

**Affiliations:** a Institute of Parasitology, Department of Pathobiology, University of Veterinary Medicine, Veterinärplatz 1, A-1210, Vienna, Austria; b Center for Emerging and Reemerging Infectious Diseases, Division of Allergy and Infectious Diseases, Department of Medicine, University of Washington, 750 Republican Street, Seattle, WA, 98109, USA; c Department of Chemistry, University of Washington, Seattle, WA, 98195, USA; d Institute of Parasitology, Vetsuisse Faculty, University of Bern, Länggasstrasse 122, 3012, Bern, Switzerland

**Keywords:** Cystoisosporosis, *Cs*CDPK1, Efficacy, Pharmacokinetics, Safety

## Abstract

Cystoisosporosis is a leading diarrheal disease in suckling piglets. With the confirmation of resistance against the only available drug toltrazuril, there is a substantial need for novel therapeutics to combat the infection and its negative effects on animal health. In closely related apicomplexan species, bumped kinase inhibitors (BKIs) targeting calcium-dependent protein kinase 1 (CDPK1) were shown to be effective in inhibiting host-cell invasion and parasite growth. Therefore, the gene coding for *Cystoisospora suis* CDPK1 (*Cs*CDPK1) was identified and cloned to investigate activity and thermal stabilization of the recombinant *Cs*CDPK1 enzyme by BKI 1369. In this comprehensive study, the efficacy, safety and pharmacokinetics of BKI 1369 in piglets experimentally infected with *Cystoisospora suis* (toltrazuril-sensitive, Wien-I and toltrazuril-resistant, Holland-I strains) were determined *in vivo* and *in vitro* using an established animal infection model and cell culture, respectively. BKI 1369 inhibited merozoite proliferation in intestinal porcine epithelial cells-1 (IPEC-1) by at least 50% at a concentration of 40 nM, and proliferation was almost completely inhibited (>95%) at 200 nM. Nonetheless, exposure of infected cultures to 200 nM BKI 1369 for five days did not induce structural alterations in surviving merozoites as confirmed by transmission electron microscopy. Five-day treatment with BKI 1369 (10 mg/kg BW twice a day) effectively suppressed oocyst excretion and diarrhea and improved body weight gains in treated piglets without obvious side effects for both toltrazuril-sensitive, Wien-I and resistant, Holland-I *C. suis* strains. The plasma concentration of BKI 1369 in piglets increased to 11.7 μM during treatment, suggesting constant drug accumulation and exposure of parasites to the drug. Therefore, oral applications of BKI 1369 could potentially be a therapeutic alternative against porcine cystoisosporosis. For use in pigs, future studies on BKI 1369 should be directed towards ease of drug handling and minimizing treatment frequencies.

## Highlights

• Oral application of BKI 1369 effectively reduced oocyst excretion and diarrhea in *Cystoisospora suis* infected piglets.
• 200 nM of BKI 1369 almost completely suppressed parasite proliferation *in vitro.*
• IC_50_ and IC_95_ concentrations of BKI 1369 did not induce morphological alterations in *in vitro* cultured merozoites.
• *Cystoisospora* *suis* CDPK1, the putative target of BKI 1369, has glycine as gatekeeper residue.

## Graphical abstract

**Figure:**
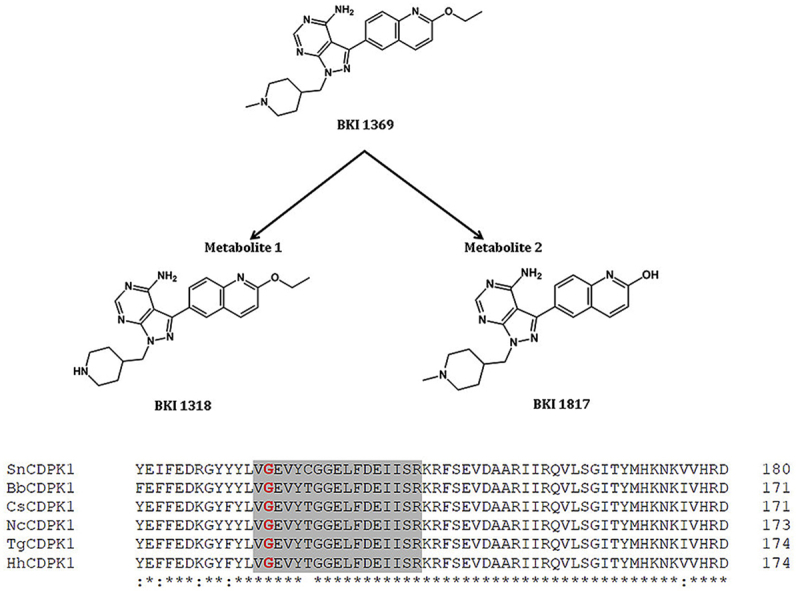

## 1 Introduction

Calcium-dependent protein kinases (CDPKs) are promising targets for the development of anti-apicomplexan drugs (Hui et al., 2015; Kugelstadt et al., 2011; Ojo et al., 2013). Apicomplexan CDPKs, belonging to a superfamily of serine-threonine kinases, are among the most abundant classes of calcium sensors and are crucial for multiple physiological functions such as gliding, cell invasion, egress and replication (Kugelstadt et al., 2011; Ojo et al., 2012). Most importantly, these CDPKs are absent in mammalian hosts, rendering them excellent parasite-specific drug targets (Billker et al., 2009; Larson et al., 2012; Ojo et al., 2010). CDPKs in apicomplexan parasites can be effectively targeted by bumped kinase inhibitors (BKIs), a class of synthetic competitive inhibitors of ATP-binding. BKI selectivity is based on a small ‘gatekeeper’ residue present in apicomplexan CDPK1 (Keyloun et al., 2014). This allows for BKIs with large aromatic moieties displayed from the C-3 position of the pyrazolopyrimidine scaffold to selectively target the apicomplexan enzyme and not mammalian or other parasite kinases.

A wide range of BKIs have already been evaluated for efficacy against coccidian parasites such as *Toxoplasma gondii* (Doggett et al., 2014; Johnson et al., 2012; Winzer et al., 2015)*, Neospora caninum* (Ojo et al., 2014; Sánchez-Sánchez et al., 2018), *Besnoitia besnoiti* (Jiménez-Meléndez et al., 2017), *Sarcocystis neurona* (Ojo et al., 2016) and *Cryptosporidium parvum* (Hulverson et al., 2017; Schaefer et al., 2016). No consistent side effects of BKIs have been observed in these *in vivo* trials. *Cystoisospora suis* is a close relative of *T. gondii* and *N. caninum* within the family Sarcocystidae (Ogedengbe et al., 2015; Samarasinghe et al., 2008). Therefore, existence of the ortholog of CDPK1 in *C. suis* and consequently efficacy of BKIs, originally developed for inhibition of *Tg*CDPK1, was expected.

*C. suis* (syn. *Isospora suis*) is one of the leading causes of diarrheal disease in suckling piglets. The disease is usually non-fatal but of high morbidity in affected litters (Lindsay et al., 1985; Roepstorff et al., 1998; Sayd and Kawazoe, 1998). Cystoisosporosis is characterized by watery-to-pasty non-hemorrhagic diarrhea, impaired weight gain and reduced and uneven weaning weights in piglets, and consequently imposes significant economic losses on the pig farming industry (Mundt et al., 2005, 2007; Shrestha et al., 2015). The triazinone toltrazuril is the only commercially marketed compound available for effective treatment of cystoisosporosis. Due to the enforcement of strict regulations on the use of anticoccidials in many countries and, most importantly, the emergence of resistance against toltrazuril in *C. suis* (Shrestha et al., 2017), the search for alternative effective therapeutic and control strategies against cystoisosporosis is a priority.

BKIs targeting apicomplexan CDPK1 have been shown to inhibit parasite infection *in vitro* and/or in representative animal models. In the present study, BKI 1369 successfully ameliorated cystoisosporosis in its natural host, the pig, employing experimental infections with two different strains of *C. suis* with varying susceptibility to toltrazuril. The *in vivo* results were further supported by *in vitro* demonstration of efficacy against *C. suis* merozoites in IPEC-1 cell cultures and genetic and functional studies on the putative target enzyme, *Cs*CDPK1. Repeated oral applications of BKI 1369 showed marked systemic exposure after treatment, significantly reduced oocyst excretion and diarrhea, and improved body weight gains in treated piglets. The results of the animal experimental and *in vitro* studies thus validate BKI 1369 as a potential therapeutic candidate against porcine cystoisosporosis, and they also have possible implications for efficacy studies of BKI 1369 against cystosiospororsis in other mammalian hosts.

## 2 Materials and methods

### 2.1 Compounds used

BKIs 1369 and 1318 (metabolite 1) were synthesized to >95% purity as assessed by high performance liquid chromatography (HPLC) as previously described (Johnson et al., 2012; Vidadala et al., 2016). BKI 1369 for the animal studies was synthesized by VAS Bio, Cherlapally, Hyderabad, India to >98% purity by HPLC and nuclear magnetic resonance (NMR) with <0.5% of any single impurity by HPLC and to less than 10 parts per million of heavy metal contamination. BKI 1817 (metabolite 2) (Hulverson et al., 2017; Lee et al., 2018) was synthesized as follows: BKI 1369 was added to concentrated hydrochloric acid and stirred for 8 h at 60 ^°^C. The reaction mixture was first neutralized and then basified (pH = 8–9) using aqueous sodium bicarbonate followed by extraction with ethyl acetate (3 × 10 ml). The organic layers were combined, concentrated by rotary evaporator and purified by reverse phase-HPLC in acetonitrile: water to yield BKI 1817 (metabolite 2). Synthesis of BKI 1817: ^1^H NMR (500 MHz, CD_3_OD) *δ* 8.47 (s, 1H), 8.07 (d, *J* = 8.9 Hz, 1H), 8.02 (s, 1H), 7.90 (d, *J* = 8.3 Hz, 1H), 7.56 (d, *J* = 8.0 Hz, 1H), 6.71 (d, *J* = 9.4 Hz, 1H), 4.50 (d, *J* = 6.2 Hz, 2H), 3.55 (m), 3.34 (s, 1H), 2.49 (m, 2H), 3.36 (s, 3H), 2.41 (s, 1H), 2.28 (m, 2H), 1.69 (m, 2H); MS (ESI) 390.3 *m/z* [MH+], C_21_H_24_N_7_O requires 389.5; HPLC purity >95%.

### 2.2 Molecular cloning, protein expression, purification and enzyme activity of *Cs*CDPK1

Amino acid sequences of relevant BKI-binding CDPK1 proteins from closely related apicomplexan species *T. gondii* [accession no. TGME49_301440], *N. caninum* [NCLIV_011980], *S. neurona* [SRCN_3314], *B. besnoiti* [KY991370] and *Hammondia hammondi* [HHA_301440] were obtained from ToxoDB (http://toxodb.org) and NCBI (https://www.ncbi.nlm.nih.gov/) and compared with amino acid sequences of all protein kinases predicted in the *C. suis* genome (GenBank^®^ accession number: PRJNA341953) using BLASTP (https://blast.ncbi.nlm.nih.gov/Blast.cgi) to identify the *C. suis* ortholog (*Cs*CDPK1). The sequences were then aligned and analyzed using Clustal Omega (Sievers et al., 2011).

The complete reading frame of *Cs*CDPK1 was amplified by PCR and cloned into the ligation independent cloning (LIC) site of protein expression vector AVA0421 (Ojo et al., 2011). Expression vector plasmid DNA was purified using the ZymoPURE Plasmid Kits (Zymo Research, Irvine, CA). A comparative analysis of the nucleotide sequence of the DNA insert to GenBank^®^ entries was used to confirm it as the putative *Cs*CDPK1 gene. Recombinant expression of *Cs*CDPK1 was performed in Rosetta Oxford *Escherichia coli* (Invitrogen, Carlsbad, CA, USA) at 20 °C using Studier auto-induction protocols (Studier, 2005). Soluble recombinant *Cs*CDPK1 enzyme was purified as described previously (Ojo et al., 2011). Recombinant enzyme activity and inhibition of *Cs*CDPK1 kinase phosphorylation properties by small molecule inhibitors were measured in a non-radioactive Kinase-Glo^®^ (Promega, Madison, WI, USA) luciferase assay, where readout in an EnVision Multilabel Plate Reader (PerkinElmer, Waltham, MA) was based on changes in ATP concentration in the presence of the peptide substrate. PLKtide (Signalchem Richmond, BC, Canada) with peptide sequence CKKLGEDQAEEISDDLLED-SLSDEDE was used as the receptor of the phosphate group donated by ATP in the *Cs*CDPK1 catalyzed phosphorylation reaction. The activity assay reaction buffer contained 20 mM 4-(2-hydroxyethyl)-1-piperazineethanesulfonic acid (HEPES) pH 7.5, 0.1% bovine serum albumin (BSA) (w/v), 10 mM MgCl_2_, 1 mM ethylene glycol-bis(β-aminoethyl ether)-N,N,N′,N'-tetraacetic acid (EGTA), with or without 2 mM CaCl_2_. The reaction mixture included 6 nM *Cs*CDPK1 and 68 μM PLKtide in 96-well plates with dilutions from 2 μM to 0.001 μM. The reactions were initiated by the addition of 1 μM ATP and incubated for 90 min at 30 °C and 90 rpm. Finally, the concentration of compound resulting in a 50% reduction in the enzyme activity was determined.

### 2.3 Thermal stability assay

Thermal stabilization of recombinant *Cs*CDPK1 in the presence or absence of inhibitors was determined in a 96-well format as previously described (Crowther et al., 2009; Ojo et al., 2016). Each 20 μL final assay volume contained a reaction mixture of 10 μM *Cs*CDPK1 enzyme, 20 μM of each inhibitor, 2.5% SYPRO Orange dye (Invitrogen, Carlsbad, CA) and 5% dimethyl sulfoxide (DMSO) in the presence or absence of calcium. The reactions were performed in a buffered solution containing 25 mM HEPES (pH 7.25), 500 mM NaCl, 5% glycerol, 1 mM dithiothreitol and 0.025% sodium azide with or without 250 μM EGTA as a calcium ion chelator. An internal control comprising of *Cs*CDPK1 and 5% DMSO was included in each assay plate. An additional control using previously characterized recombinant *Tg*CDPK1 enzyme was also included. The assay was performed in a StepOnePlus™ Real-Time PCR system (Applied Biosystems). All assays were performed independently at least two times (Ojo et al., 2016).

### 2.4 *In vitro* culture of intestinal porcine epithelial cells and viability assays

Intestinal porcine epithelial cells-1 (IPEC-1; DSMZ, ACC 705; www.dsmz.de) were maintained in culture medium at 37 °C and 5% CO_2_ (DMEM/HAM12 supplemented with 5% fetal calf serum and penicillin/streptomycin; Gibco via Thermofisher, Vienna, Austria) as described earlier (Worliczek et al., 2013). BKI 1369 was stored as a 20 mM stock solution in 100% DMSO at −20 °C. Cell viability in the presence of DMSO and BKI 1369 was determined by a colorimetric cell proliferation WST-1 assay (Roche Diagnostics GmbH, Mannheim, Germany) according to the manufacturer's protocol. IPEC-1 cells in 48-well plates (4 × 10^4^ cells/well) were incubated one day after seeding with different concentrations of BKI 1369 (1000 nM, 200 nM, 100 nM, 50 nM and 25 nM diluted in culture medium and 0.005% or 0.001% of DMSO) in quadruplicate and grown for 4 days at 37 °C with 5% CO_2_ in a humidified incubator. After cultivation, 10 μl of WST-1 reagent was added to each well and incubated for 3 h at 37 °C. Absorbance was measured at 409 nm (reference wavelength 620 nm) using an ELISA plate reader (Filter-Max F5, Molecular Devices, Sunnyvale, CA, USA). Cell viability was compared against DMSO-only and medium-only controls.

### 2.5 *In vitro* efficacy testing

Confluent IPEC-1 cells (4 × 10^4^ cells/well) grown in 48 well plates were infected with excysted sporozoites of *C. suis* (Wien-I) as described earlier (Worliczek et al., 2013) one day after cell seeding in a ratio of 80:1. Infected cells were incubated in the presence of BKI 1369 (200, 100, 50, 25, and 12.5 nM) in culture medium continuously for 5 days (0–4 days post infection (dpi)) to calculate the 50% and 95% inhibitory concentrations (IC_50_ and IC_95_). After treatment for 5 days (0–4 dpi), medium containing BKI 1369 was replaced with normal culture medium and the culture was maintained further until 9 dpi. To evaluate growth of the parasite, supernatants were collected and free merozoites were counted in pooled samples at 9 dpi from quadruplicate wells in C-Chip disposable hemocytometers (NanoEnTek/Roth Lactan, Graz, Austria). All assays included medium-only and DMSO-only controls in quadruplicate.

### 2.6 Transmission electron microscopy

Confluent IPEC-1 cells (2.5 × 10^5^ cells/well) grown in 6-well culture plates (VWR, Vienna, Austria) were infected with 5 × 10^3^ *C. suis* sporozoites. The next day, cells were incubated with predetermined IC_50_ (40 nM) or IC_95_ (200 nM) concentrations of BKI 1369 (see above section on efficacy testing) at 40 °C for five days. Infected cells incubated with 0.001% DMSO served as controls. After 24 h, 72 h, 96 h and 120 h of incubation, medium supernatant was removed, the monolayers were washed twice with phosphate buffer saline (PBS) and fixed with 5% glutaraldehyde (Merck, Darmstadt, Germany) in 0.1 M sodium phosphate buffer (pH 7.2) for 10 min at 4 °C. Cells were carefully collected using a rubber scraper (TPP, Switzerland) and subjected to centrifugation at room temperature for 5 min at 390×*g*. The supernatant was removed and cells were postfixed with 1% osmium tetroxide (Merck) for 1 h at 4 °C. After dehydration in an alcohol series and propylene oxide (Merck), the cells were embedded in glycidyl ether 100 (Serva, Heidelberg, Germany). The ultra-thin sections were cut on a Leica Ultramicrotome (Leica Ultracut S, Vienna, Austria), stained with uranyl acetate (Sigma-Aldrich, Vienna, Austria) and lead citrate and examined in a Zeiss TEM 900 electron microscope (Carl Zeiss, Oberkochen, Germany) operated at 50 kV.

### 2.7 Study animals and husbandry

A total of 35 conventionally raised piglets from four crossbred sows (Landrace x Large White) were randomly allocated to four treatment groups (Table 1). Pregnant sows were moved to the animal husbandry facility of the Institute of Parasitology, University of Veterinary Medicine Vienna, Austria, two weeks before farrowing to acclimatize to the housing conditions. Sows were housed on straw in individual farrowing crates and fed once daily with a commercial feed free of coccidiostat. The piglets received milk from the sow followed by starter feed from the second week of life. Fresh drinking water was provided *ad libitum* to the sows and piglets. Only clinically healthy piglets with a body weight ≥900 g on study day 1 (SD 1) were included in the study. The first day after the birth of piglets was considered SD 1. The clinical study lasted for 29 days (SD 29) and followed a blinded and randomized experimental block design with the individual piglet as an experimental unit.

**Table 1:** Litters and treatment groups used in the trial; piglets were infected on study day 3 and treated with BKI 1369 from study day 3–7.

Group	Infection with *C. suis* strain	Litter No.	No. of piglets in total	No. of piglets that completed the study^c^	Treatment
Wien-BKI	Wien-I	1, 2, 3	13	10	BKI; 10 mg/kg BW, twice daily
Wien-Ctrl	Wien-I	1, 2, 3	12	9	Mock^a^; twice daily
Holl-BKI	Holland-I	4	5	5	BKI; 10 mg/kg^b^ BW, twice daily
Holl-Ctrl	Holland-I	4	5	5	Mock^a^; twice daily^§^

a Mock: solvent (3% Tween 80 + 7% ethanol + 90% 0.9% NaCl).

b Piglets infected with Holland-I strain (Holl-BKI & Holl-Ctrl) were additionally treated with 20 mg/kg BW toltrazuril.

c Three piglets from group Wien-BKI and two piglets from group Wien-Ctrl were sacrificed on SD 9 for collection of samples for histopathology. In addition, one piglet from group Wien-Ctrl was found dead on SD 11. Only those piglets that completed the study were included in the analysis of efficacy.

All the procedures involving animals were approved by the Animal Ethics Committee of the University of Veterinary Medicine Vienna and the national authority according to § 26ff of Animal Experiments Act, Tierversuchsgesetz 2012-TVG 2012 (license number: BMWF-68.205/0034-WF/V/3b/2016; Austrian Federal Ministry of Science, Health and Economy).

### 2.8 *C. suis* oocysts, infection and BKI 1369 treatment

A toltrazuril-sensitive laboratory strain of *C. suis*, Wien-I (Joachim and Mundt, 2011) and a toltrazuril-resistant field isolate of *C. suis*, Holland-I (Shrestha et al., 2017), were maintained and passaged regularly in suckling piglets every 3–6 months for the production of infectious oocysts at the Institute of Parasitology, University of Veterinary Medicine Vienna, Austria. To avoid unintended mixing of the two strains, all procedures (strain maintenance and experimental infections) were carried out in separated trials with complete cleaning and disinfection of the farrowing units between trials. Oocysts of *C. suis* were isolated from fecal samples and purified as described earlier (Worliczek et al., 2013). For sporulation, purified oocysts in 2% aqueous potassium dichromate solution were poured into petri dishes, aerated twice daily and incubated at room temperature for 2–3 days. The sporulated oocysts were stored in 2% aqueous potassium dichromate solution at 11 °C until used.

Each piglet was orally infected on SD 3 (for experimental setup, see Table 1) with a single dose of approximately 1000 sporulated oocysts of the respective *C. suis* strain using a flexible plastic Pasteur pipette. Immediately after infection, piglets were either treated with BKI 1369 or mock treated with solvent (3% Tween 80 + 7% ethanol + 90% 0.9% NaCl) twice daily for five days from SD 3 to SD 7. Fine powder of BKI 1369 was dissolved in the solvent to yield a 2.5% BKI solution, which was used to treat individual piglets in groups Wien-BKI and Holl-BKI twice daily at the rate of 10 mg/kg body weight (BW) (0.4 ml 2.5% BKI 1369/kg BW). Furthermore, piglets infected with the Holland-I isolate of *C. suis* (Holl-BKI and Holl-Ctrl) were additionally treated with 20 mg/kg BW of toltrazuril (Baycox^®^ 5%, Bayer Animal Health, Monheim, Germany) to assure that only toltrazuril-resistant parasites were present.

### 2.9 Post-treatment observations

Post-treatment observations were conducted by a veterinarian immediately after the end of each treatment and 2 h post-treatment to account for any adverse events associated with the treatment. All piglets were examined post-treatment for systemic or regional responses such as abnormal behavior, salivation/vomiting, ocular discharge, abnormal locomotion etc. In addition, all piglets were observed daily during the course of the studies to ensure good general health and any condition that required veterinary care was recorded and addressed.

### 2.10 Sample collection and efficacy parameters

The efficacy of BKI 1369 was evaluated by the assessment of oocyst excretion, fecal consistency and body weight development. Individual fecal samples were collected daily from SD 7 to SD 20 for the evaluation of fecal consistency and oocyst excretion. Fecal consistency was scored immediately after sampling with fecal scores (FS) 1: normal/firm, FS 2: pasty, FS 3: semi-liquid and FS 4: liquid, with FS 3 and FS 4 considered as diarrhea. Fecal samples were screened for the presence of oocysts qualitatively by autofluorescence (AF) followed by a quantitative modified McMaster technique (Joachim et al., 2018b). Furthermore, pooled fecal samples of each litter (SD 7) were examined for the presence of other entero-pathogens such as rotavirus, coronavirus, *Escherichia coli*, and *Clostridium perfringens*. The body weight of each piglet was recorded on SD 1, 8, 15, 22, 29 and additionally on the days of treatment for calculation of the treatment doses.

Three piglets from group Wien-BKI and two piglets from group Wien-Ctrl were sacrificed on SD 9 after the confirmed onset of oocyst excretion in the mock treated group to collect jejunal tissue sections for histopathology and transmission electron microscopy (TEM). In addition, jejunal mucosal scrapings were collected from each sacrificed piglet. At the end of the experiment (SD 29), all remaining piglets from groups Wien-BKI, Wien-Ctrl, Holl-BKI and Holl-Ctrl were sacrificed.

**Fig. 1:**
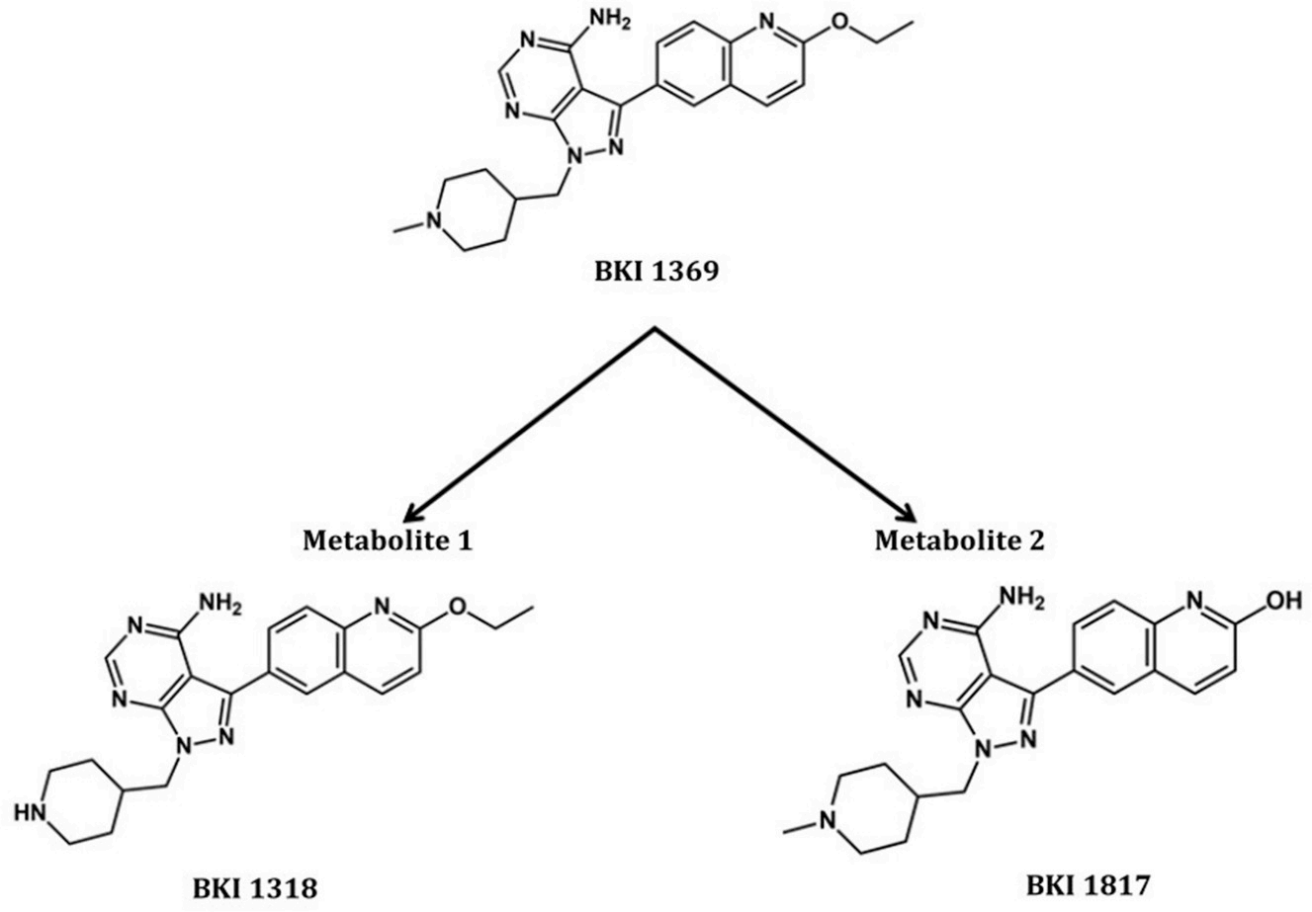
Chemical structure of bumped kinase inhibitor 1369 (BKI 1369) and its two major metabolites, BKI 1318 (metabolite 1) and BKI 1817 (metabolite 2).

Blood samples with EDTA were collected from piglets in the group Wien-BKI just before the first treatment, 2 h after the first treatment, just before the 9th treatment, 2 h after the 9th treatment and at the end of the study (SD 29). Plasma samples were obtained by centrifugation (10 min at 1500×*g*) and stored at −80 °C until use. All remaining piglets in group Wien-BKI (n = 10) were sacrificed at the end of the study and urine samples were collected. To determine compound exposure, BKI 1369 and its metabolites, BKI 1318 (metabolite 1) and BKI 1817 (metabolite 2) (Fig. 1) (Hulverson et al., 2017; Lee et al., 2018), were extracted from the plasma and urine samples using acetonitrile/0.1% formic acid with an internal standard. A standard curve was prepared for comparison and quantification. The samples were analyzed by LC-MS/MS analysis on an ACQUITY ultra performance liquid chromatography (UPLC) system in tandem with a Waters Xevo TQS Micro (Waters Corporation, Milford, MA, USA).

### 2.11 Histopathology

At necropsy, jejunal mucosal scrapings and tissue sections were collected from sacrificed piglets on SD 9. Jejunal sections for histopathology were fixed in 10% neutral buffered formalin, embedded in wax, processed, sectioned and stained with hematoxylin and eosin (H&E) at the Institute of Pathology and Forensic Veterinary Medicine, University of Veterinary Medicine Vienna, Austria. Air-dried mucosal scrapings from the jejunum were fixed in methanol and stained with Giemsa using standard procedures. Both H&E tissue sections and Giemsa stained mucosal smears were examined for the presence of developmental stages of *C. suis* at magnifications of 100× to 1000x.

### 2.12 Statistical analysis

Statistical calculations for *in vivo* trials were performed with RStudio version 1.1.453 (RStudio Team, 2018), descriptive statistics with Microsoft Excel 2010 (Microsoft Inc., Vienna, Austria) and GraphPad Prism version 5.04 for Windows (GraphPad Software, San Diego, California USA). Differences in clinical and parasitological parameters between groups were analyzed with ANOVA in the case of normal distribution and variance homogeneity of the data, or a Kruskal-Wallis rank sum test if this was not the case. In the event of significance for the omnibus tests parametric or non-parametric *post-hoc* tests for multiple comparisons were performed (according to Tukey and Conover, respectively), with *P*-value adjustment after Bonferroni. *In vitro* assays were evaluated by t-tests in GraphPad Prism version 5.04 for Windows, with significance reported at *P* < 0.05. For testing efficacy of BKI 1369 *in vitro*, the percentage of inhibition was calculated as follows:

$$\text{\%}\;\text{inhibition}=(1-\;\frac{No.\;\;of\;free\;merozoites\;counted\;in\;BKI\;1369\;treated\;culture\;at\;9\;dpi}{No.\;\;of\;free\;merozoites\;counted\;in\;DMSO-only\;treated\;culture\;at\;9\;dpi})\;x\;100$$

## 3 Results

### 3.1 Expression of recombinant *Cs*CDPK1

**Fig. 2:**
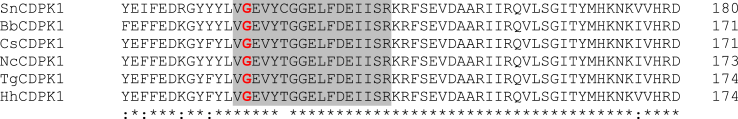
Alignment of amino acid sequences of *Sn*CDPK1, *Bb*CDPK1, *Cs*CDPK1, *Nc*CDPK1, *Tg*CDPK1 and *Hh*CDPK1. The ATP binding region is shaded (grey); Gatekeeper residue ‘glycine’ is marked in red. (For interpretation of the references to color in this figure legend, the reader is referred to the Web version of this article.)

Comparative sequence analysis of the BKI-binding CDPKs in *T. gondii* (*Tg*CDPK1), *N. caninum* (*Nc*CDPK1), *S. neurona* (*Sn*CDPK1), *H. hammondi* (*Hh*CDPK1) and *B. besnoiti* (*Bb*CDPK1) against the predicted protein kinases of *C. suis* identified CSUI_000151 as the putative CDPK1 ortholog (Fig. 2). This CDPK1-type kinase in *C. suis* (*Cs*CDPK1) showed a high degree of sequence similarity in the ATP binding domain with >97% amino acid identity to the above mentioned apicomplexan parasites including glycine as the gatekeeper residue.

**Supplementary Fig. S1:**
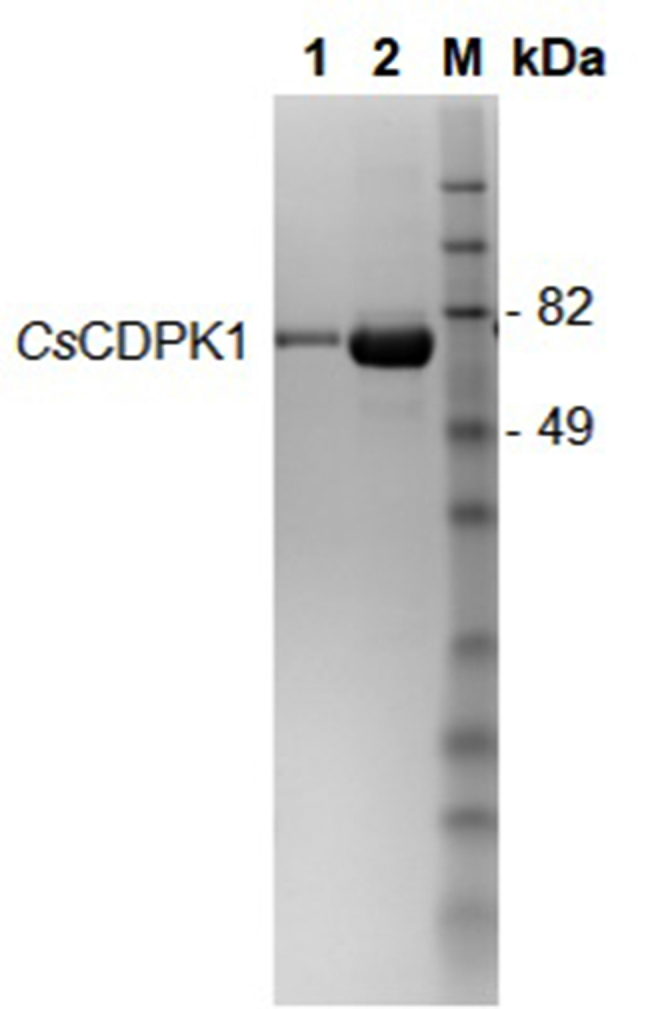
Coomassie blue-stained SDS-PAGE gel of recombinant CsCDPK1 protein; M: molecular weight standards; Lane 1: IMAC elution 1 of CsCDPK1; Lane 2: IMAC elution 2 of CsCDPK1; IMAC: immobilized metal ion affinity chromatography

*Cs*CDPK1 was expressed in *E*. *coli.* Analysis of the recombinant expression by SDS-PAGE showed a protein band which matched the predicted molecular weight of 60 kDa for the uncleaved, His-tagged *Cs*CDPK1 (Supplementary Fig. S1).

### 3.2 Functional assessment of *Cs*CDPK1 in the presence of BKI 1369

**Supplementary Fig. S2:**
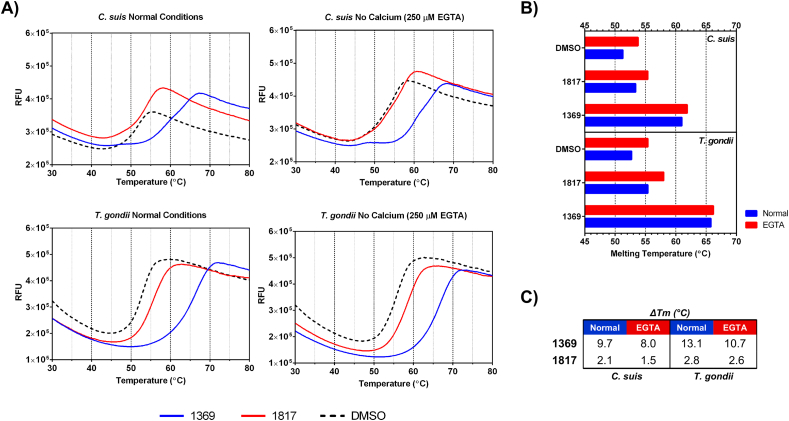
Thermal melt curves of recombinant CsCDPK1 and TgCDPK1 with BKIs in the presence (Normal Conditions) or absence (250 μM EGTA) of calcium (A); Melting temperatures as quantified by Boltzmann sigmoidal analysis (B); Shift in melting temperatures (ΔTm) from DMSO controls (C)2

Recombinant *Cs*CDPK1 was characterized by thermal stability assays. Substantial shifts in melting temperature were observed in the presence of BKI 1369 (9.7 °C and 8.0 °C in the presence of EGTA) relative to 1817 (metabolite 2) (2.1 °C and 1.5 °C in the presence of EGTA) and the no compound DMSO control (Supplementary Fig. S2A-C). Recombinant *Cs*CDPK1 was found to have protein kinase activity by transferring a phosphate group from ATP to PLKtide. The concentration of BKIs required for 50% inhibition of *Cs*CDPK1 enzyme activity (IC_50_) was in the lower nanomolar range for BKI 1369 (4.5 nM), while BKI 1817, (metabolite 2), used as a negative control, had an IC_50_ value of >481 nM.

### 3.3 Viability of IPEC-1 cells in presence of BKI 1369

**Supplementary Fig. S3:**
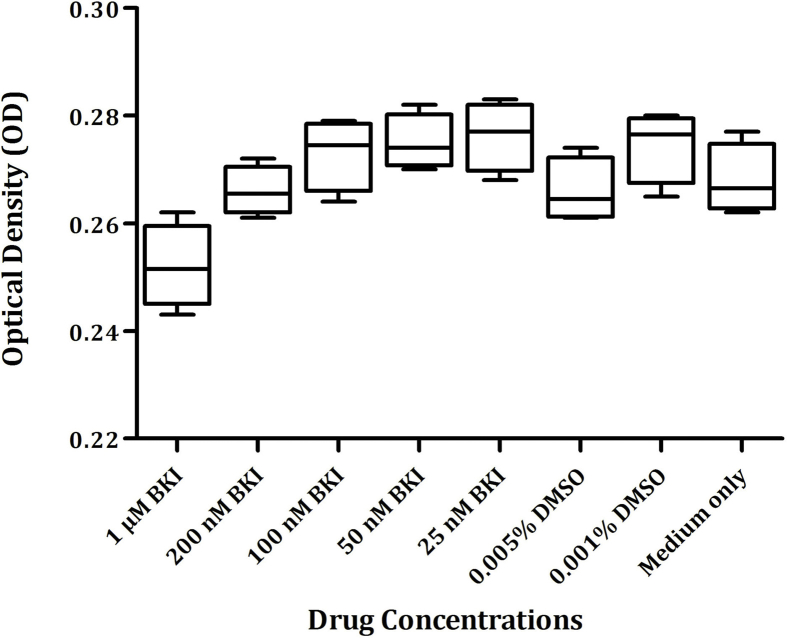
Viability and proliferation of IPEC-1 cells under different concentrations of BKI 13693

An *in vitro* assay was established to assess viability of IPEC-1 cells upon exposure to BKI 1369. Incubation of uninfected cells with BKI 1369 at concentrations <1 μM did not cause significant changes in cell proliferation and viability (Supplementary Fig. S3) compared with that of medium-only and DMSO-only controls (*P* > 0.05). Moreover, no alterations in morphology of IPEC-1 cells were detected upon inspection by light microscopy. Incubation of cells with 1 μM BKI 1369 for 4 days significantly reduced the absorbance of WST-1, directly proportional to cell viability, when compared to medium-only (*P* = 0.0196) and DMSO-only (*P* = 0.0046) controls. Consequently, the highest concentration of BKI 1369 used in subsequent culture assays was 200 nM.

### 3.4 Dose-response experiments

**Fig. 3:**
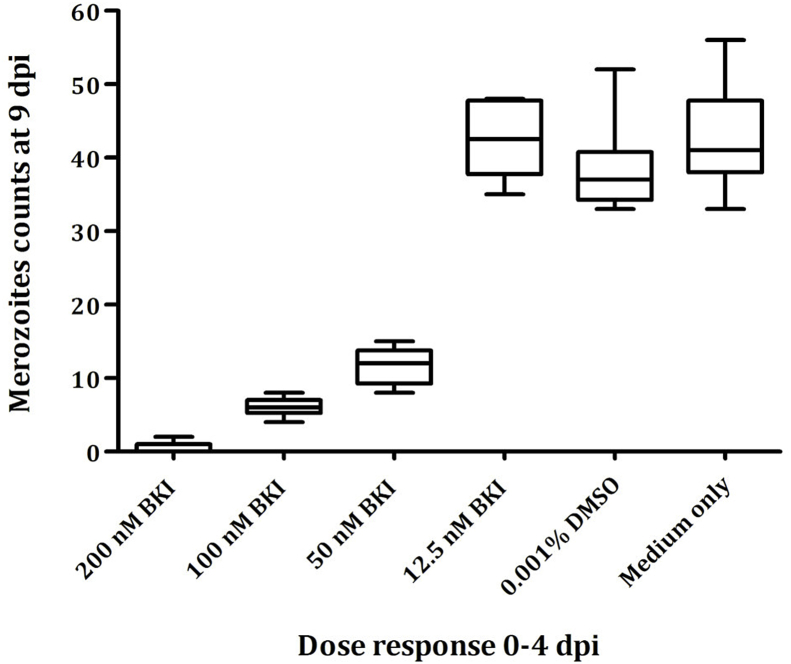
*In vitro* dose-dependent growth inhibition by BKI 1369; Cultures were infected with Wien-I *C. suis* strain and treated immediately following infection to 4 dpi.

BKI 1369 inhibited the proliferation of *C. suis* merozoites cultured in IPEC-1 cells with an IC_50_ of 40 nM, and parasite proliferation was almost completely inhibited (>95%) at 200 nM. Incubation of host cells immediately after infection up to 4 dpi resulted in a significant dose-dependent reduction of free merozoite counts at 9 dpi for all tested concentrations of BKI 1369 higher than 12.5 nM (*P* < 0.0001) (Fig. 3).

### 3.5 Light microscopy and TEM of *in vitro* samples

**Fig. 4:**
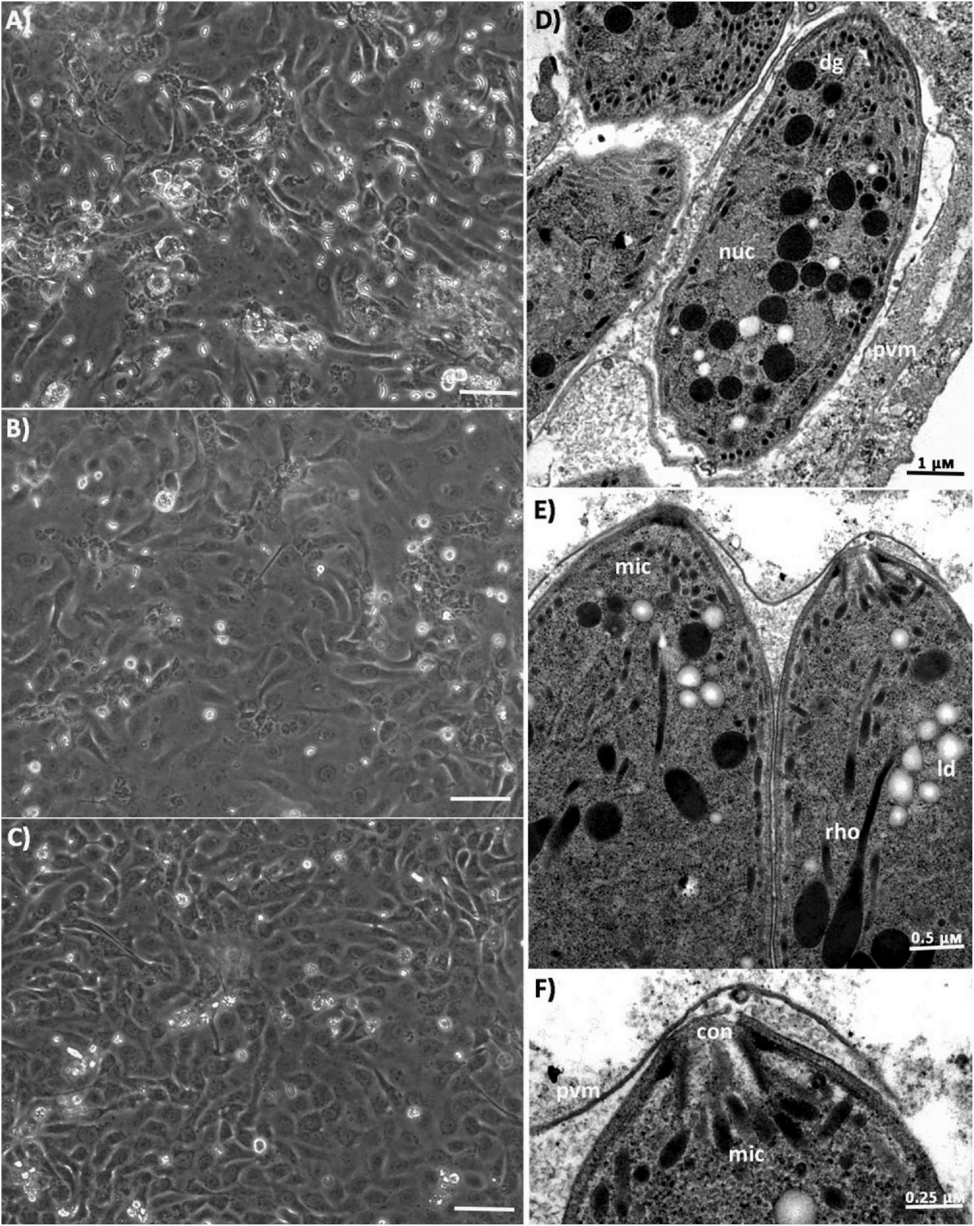
Light microscopy and TEM analysis of BKI 1369 untreated and treated *C. suis* infected IPEC-1 monolayers 5 dpi; Compared to control culture (A) significantly lower number of free merozoites counts were observed in cultures treated with IC_50_ (B) and IC_95_ (C) concentrations of BKI; Transmission electron micrograph of *C. suis* merozoites in untreated (D) and BKI treated (E & F) cultures; con: conoid; rho: rhoptries; mic: microneme; dg: dense granule; nuc: nucleus; ld: lipid droplet; pvm: parasitophorus vacuole membrane.

The effects of continued BKI 1369 treatment with IC_50_ and IC_95_ concentrations on *C. suis* propagated in IPEC-1 cells were visualized by light microscopy. In BKI 1369 treated cultures, marked reductions in the number of intracellular and free merozoites were observed (Fig. 4A–C). Intracellular and free merogonic stages were frequently observed in DMSO-only control cultures under a light microscope compared to treated cultures. However, these concentrations had no apparent effects on the ultrastructure of *C. suis* merozoites as analyzed by TEM (Fig. 4D–F). Although fewer intracellular merozoites were observed in longitudinal sections of treated cultures, they were confined within a parasitophorous vacuole, surrounded by a parasitophorus vacuole membrane and exhibited typical features of all apicomplexans including the apical complex with spirally arranged conoid, micronemes, rhoptries and dense granules (Fig. 4D–F).

### 3.6 Oocyst excretion

**Fig. 5:**
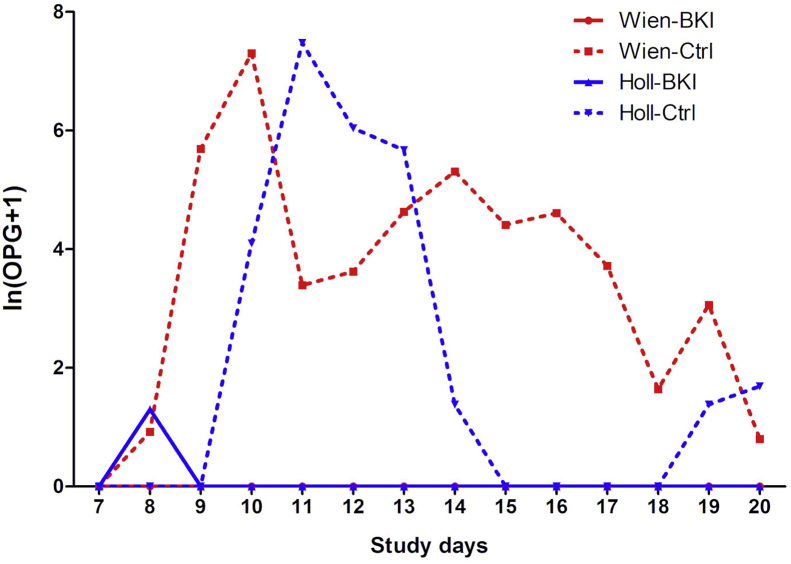
Daily oocyst excretion ln (OPG+1) per group during the whole study period as determined by McMaster technique.

Treatment with BKI 1369 completely suppressed oocyst excretion in the group Wien-BKI and significantly limited excretion in group Holl-BKI to a single positive sample on SD 8 (Fig. 5). In contrast, oocyst shedding was observed in both mock treated control groups, Wien-Ctrl and Holl-Ctrl, as early as SD 8, and by SD 12 all piglets in these groups had excreted oocysts at least once. The mean duration of oocyst excretion was 5.3 ± 3.8 days in the group Wien-Ctrl and 2.8 ± 1.3 days in the group Holl-Ctrl (Supplementary Table S1). All parameters related to oocyst excretion such as the number of days with McMaster detectable oocyst excretion, the mean number of oocysts per grams of feces (OpG) and the area under the curve (AUC) for OpG were significantly different between the treated and control groups, irrespective of the *C. suis* strain used (Table 2).

**Table 2:** *P* values (given as –log10) for parameters of oocysts excretion and fecal score. Differences at *P* < 0.05 are indicated in bold. OpG: oocysts per grams of feces; FS: fecal score.

Parameter	Wien-BKI *vs* Wien-Ctrl	Holl-BKI *vs* Holl-Ctrl	*Χ*^*2*^; *df*
Area under the curve OpG	**8.82**	**5.03**	23.79; 3
Mean OpG	**8.72**	**2.36**	33.13; 3
Days with McMaster countable oocysts excretion	**8.56**	**4.23**	23.40; 3
Mean fecal score	**3.74**	0.62	18.52; 3
Area under the curve FS	**3.49**	**3.11**	18.93; 3
Number of diarrhea days	**3.72**	**2.99**	20.00; 3

### 3.7 Fecal consistency and diarrhea

**Fig. 6:**
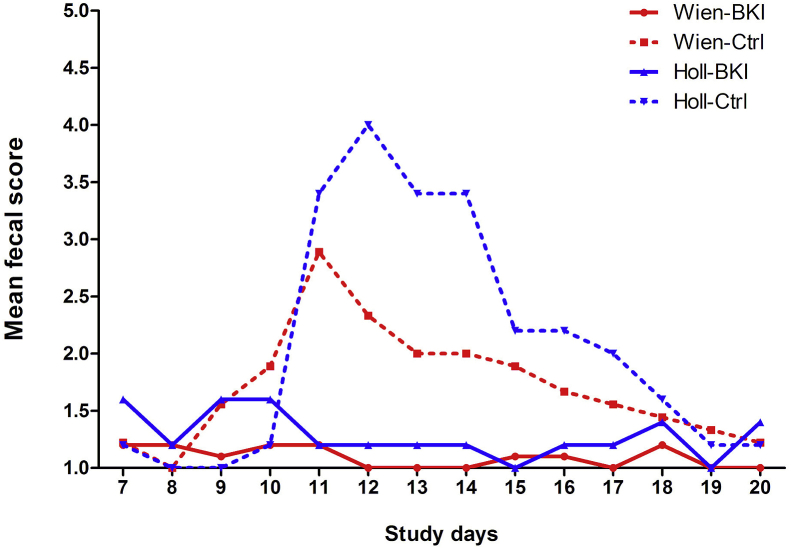
Mean fecal scores of piglets throughout the sampling period of 14 days.

None of the piglets had diarrhea on the day of infection. The mean daily FS in group Wien-BKI did not exceed 1.2 throughout the study and was significantly lower than that of Wien-Ctrl (Fig. 6, Table 2). Three samples in group Holl-BKI had a FS 3, but the FS in group Holl-BKI was significantly lower than that of the Holl-Ctrl group throughout the study (Table 2). The maximum prevalence of diarrhea was 100% in group Holl-Ctrl (average duration 4.4 days) and 66.7% in group Wien-Ctrl (average duration 2.4 days). Watery diarrhea (FS 4) was observed in 33.3% and 100% of the piglets in groups Wien-Ctrl and Holl-Ctrl for an average of 1.3 and 2.4 days, respectively.

### 3.8 Body weight

**Fig. 7:**
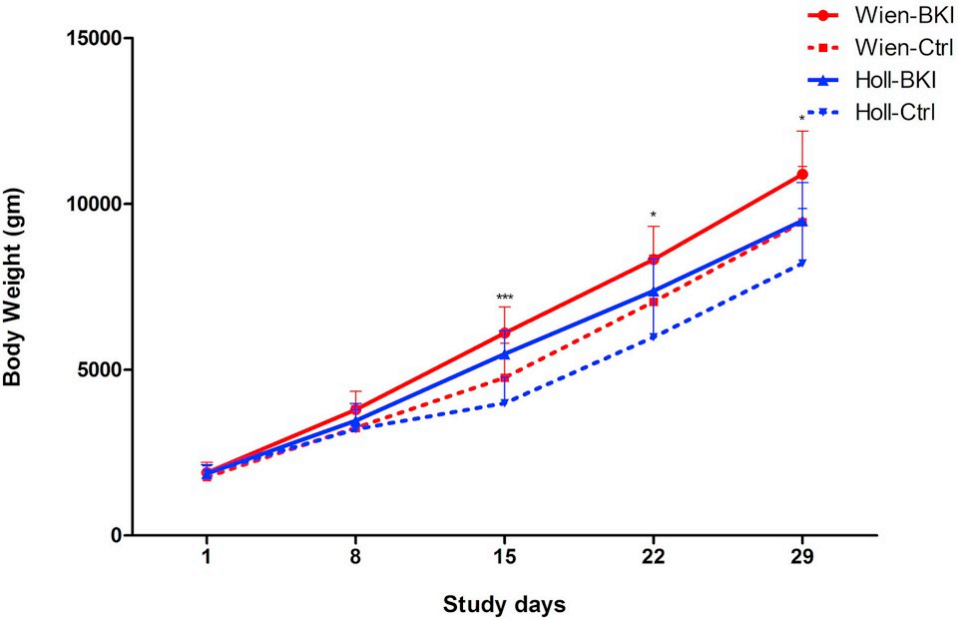
Body weight development in piglets after BKI 1369 treatment. Vertical lines depict standard deviations.

Body weights were not significantly different between the groups on SD 1, the day of randomization (*P* = 0.79). Daily weight gain and total weight gain from SD 1 to SD 29 were significantly higher in the group Wien-BKI compared to Wien-Ctrl, whereas no such differences were found between treated and control piglets infected with *C. suis* Holland-I (Table 3). During the acute phase of infection (SD 15), mean body weights were significantly higher in BKI-treated piglets compared to the control groups, irrespective of the *C. suis* strains used (Fig. 7). Also, a significant negative correlation between the mean fecal score and the total weight gain was observed for all piglets (*ρ* = −0.518, *P* = 0.02).

**Table 3:** Body weight development and body weight gains (in grams) with standard deviations in brackets. Differences at *P* < 0.05 are indicated in bold. SD: Study day.

Group	SD 1	SD 8	SD 15	SD 22	SD 29	Total BWG SD 1- SD 29	Daily weight gain
Wien-BKI	1898 [309.0]	3794 [550.9]	6102 [785.1]	8324 [995.8]	10896 [1298.0]	8998 [1158.1]	321.3 [41.3]
Wien-Ctrl	1762.2 [347.9]	3242.2 [670.3]	4755.5 [1042.1]	7042.2 [1405.6]	9446.6 [1678.6]	7684.4 [1410.4]	274.4 [50.3]
*P*	0.38	0.06	**0.005**	**0.03**	**0.04**	**0.03**	**0.03**
Holl-BKI	1868 [267.8]	3464 [517.7]	5480 [698.7]	7376 [968.4]	9492 [1145.9]	5536 [349.6]	272.2 [33.5]
Holl-Ctrl	1880 [223.1]	3200 [589.2]	3988 [783.2]	5976 [1295.6]	8204 [1652.4]	4649.6 [568.0]	225.8 [53.7]
*P*	0.94	0.47	**0.01**	0.08	0.1	0.1	0.1

### 3.9 Differential diagnosis

Fecal samples pooled by litter on SD 7 were negative for rotavirus and coronavirus whereas *E. coli* and *Cl. perfringens* could be detected in all litters.

### 3.10 Safety

No piglets showed treatment-related adverse effects that required veterinary intervention throughout the study period. Neither visible signs of toxicity nor clinically relevant systemic or regional/local responses related to treatment were observed in the treated groups.

### 3.11 Histopathology of jejunal sections and mucosal scrapings

**Fig. 8:**
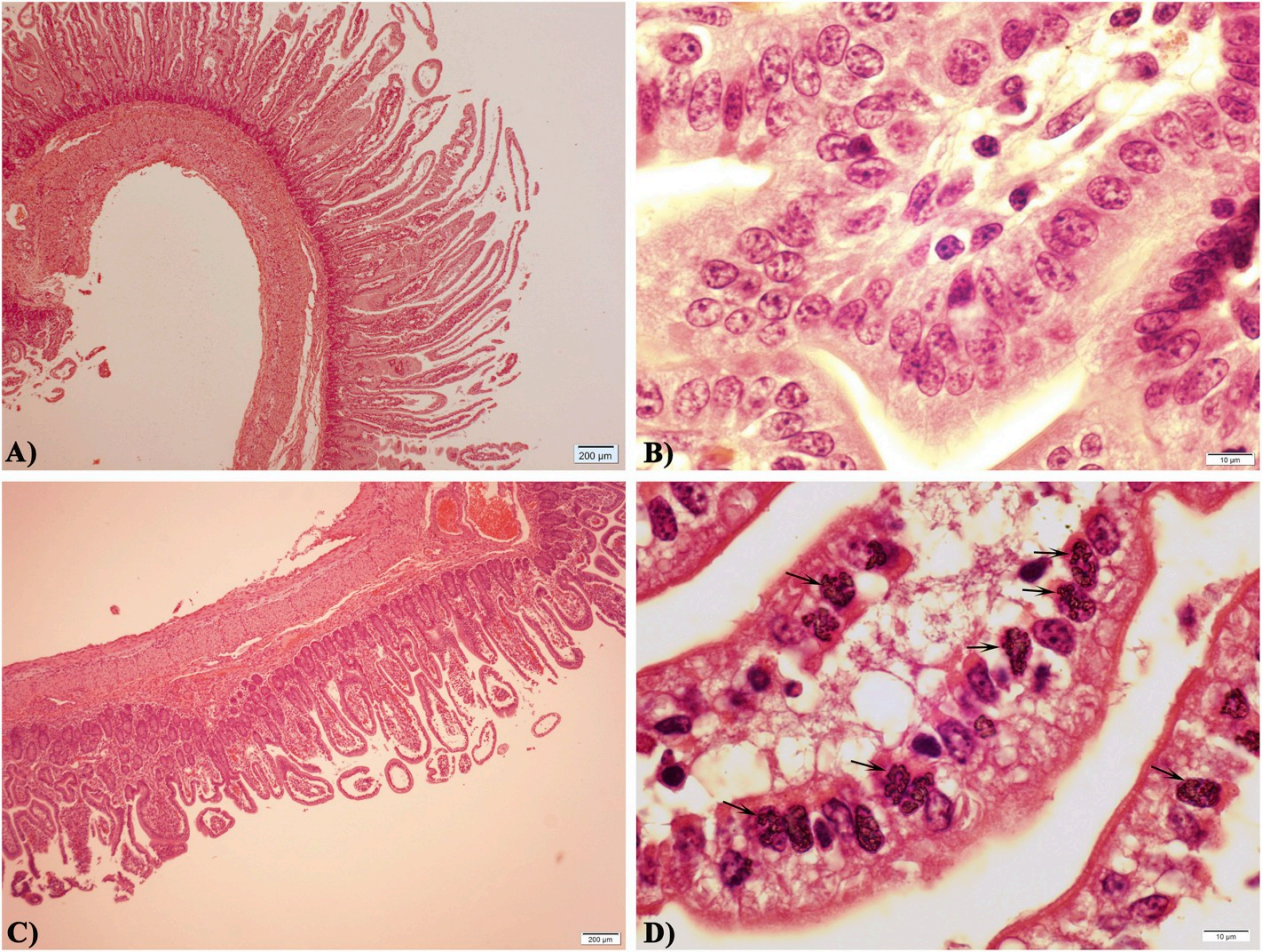
Histological sections of jejunum in *C. suis* infected 9-days-old piglets from group Wien-BKI (A–B) and Wien-Ctrl (C–D); Infection with *C. suis* resulted in stunted and atrophied villi (C) with numerous asexual endogenous stage (arrows, D); (H&E stain).

**Fig. 9:**
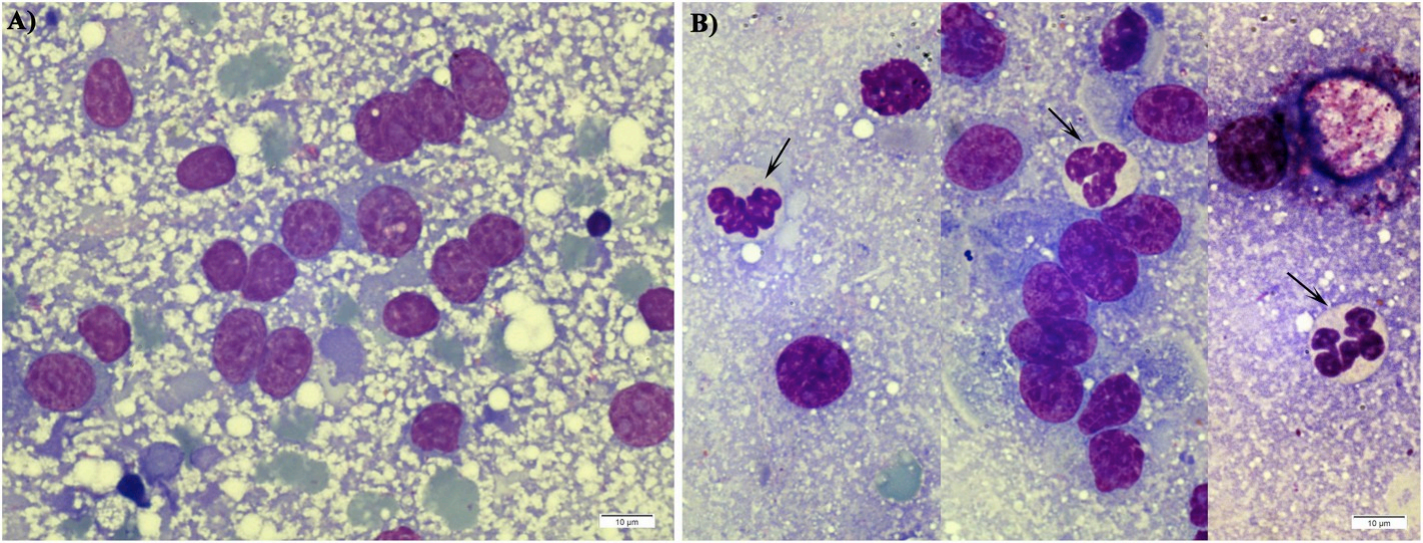
Giemsa stained mucosal scraping smears in *C. suis* infected piglets from group Wien-BKI (A) and Wien-Ctrl (B); Merozoites within parasitophorus vacuoles could be observed in smears from group Wien-Ctrl (arrows, B).

H&E stained sections of the mid-jejunum were examined for histopathological changes characteristic of *C. suis* infections and for the presence of developmental stages of the parasite. Compared to the normal jejunal villi in BKI-treated piglets, mock treated control piglets exhibited stunted villi atrophy and fusion (Fig. 8 A and C). Numerous infected epithelial cells with distinct clustered merozoites of *C. suis* were observed in stained jejunal sections of mock treated control piglets (Fig. 8D). Giemsa-stained mucosal scraping smears from mock treated piglets revealed the presence of matured crescent-shaped merozoites within the parasitophorous vacuole (Fig. 9). Neither histopathological alterations in the morphology of villi nor presence of endogenous stages of *C. suis* could be detected in tissue sections and mucosal scraping smears of BKI-treated piglets.

### 3.12 Pharmacokinetics

BKI 1369 and its metabolites BKI 1318 (metabolite 1) and BKI 1817 (metabolite 2) (Hulverson et al., 2017; Lee et al., 2018) were measured in plasma and urine samples (Table 4). BKI 1369 concentrations of 4.9 μM were observed at 2 h after the 1st dose and rose to 11.7 μM 2 h before the 9th dose, without further increase 2 h after the 9th dose (10.2 μM). Metabolite levels remained low at all measured time points and there was no compound detected in plasma or urine at 29 dpi.

**Table 4:** Concentrations of BKI 1369 and its metabolites, BKI 1318 (metabolite 1) and BKI 1817 (metabolite 2) in piglets infected with Wien-I strain; dpi: days post-infection.

Sample type and time of collection	No. of samples	Drug concentration (μM)		
		BKI 1369	BKI 1318	BKI 1817
**Plasma**				
2 dpi (0 h before 1st dose)	6	0 ± 0	0 ± 0	0 ± 0
3 dpi (2 h after 1st dose)	6	4.9 ± 2.0	0.01 ± 0.01	0 ± 0
7 dpi (2 h before 9th dose)	6	11.7 ± 4.8	0.2 ± 0.1	0.2 ± 0.01
7 dpi (2 h after 9th dose)	6	10.2 ± 2.9	0.1 ± 0.1	0.02 ± 0.01
29 dpi (termination)	6	0 ± 0	0 ± 0	0 ± 0
**Urine**				
29 dpi (termination)	5^a^	0 ± 0	0 ± 0	0 ± 0

a Urine sample of one piglet could not be obtained.

## 4 Discussion

The role of CDPKs and their potential as molecular targets for chemotherapeutic intervention in apicomplexan parasites has previously been studied in parasites of different taxonomic groups. Apicomplexan CDPK1 regulates the calcium-dependent pathway of microneme secretion, facilitates gliding locomotion and governs host cell attachment, invasion and egress (Billker et al., 2009; Carruthers and Sibley, 1999; Kieschnick et al., 2001; Wei et al., 2013). Contrary to most mammalian kinases, the presence of glycine as a gatekeeper residue in the ATP-binding site of apicomplexan CDPK1 enables BKIs to fit perfectly into a small hydrophobic pocket, rendering them highly selective and promising compounds for therapeutic intervention against these parasites.

The identified CDPK1-type kinase in *C. suis*, *Cs*CDPK1, showed a high degree of sequence similarity in the ATP binding domain with *Tg*CDPK1, *Hh*CDPK1, *Bb*CDPK1, *Sn*CDPK1 and *Nc*CDPK1 having >97% amino acid identity with glycine as the gatekeeper residue. Unlike all the previously studied apicomplexan recombinant CDPK enzymes, the peptide substrate syntide-2 (PLARTLSVAGLPGKK) was not compatible for measurement of *Cs*CDPK1 kinase activity *in vitro*. This is inspite of the highly conserved amino acid sequence similarities between *Cs*CDPK1 and other CDPK1 enzyme active sites. However, *Cs*CDPK1 auto-phosphorylation activity could be measured in the presence or absence of calcium and with or without a suitable peptide substrate. Experimental analysis confirmed its calcium dependency as determined in phosphorylation reactions using EGTA as a Ca^2+^ chelator in the presence or absence of PLKtide (Signalchem Richmond, BC, Canada) peptide substrate (CKKLGEDQAEEISDDLLED-SLSDEDE). This calcium dependent regulation of enzyme activity is a verification that it belongs to the same family as previously described apicomplexan CDPKs (Van Voorhis et al., 2017). Future studies will be needed to determine the potential divergence of functions, natural substrates or pathways of *Cs*CDPK1 relative to other CDPKs. Nonetheless, sensitivity to BKIs by a direct inhibition of enzyme activity was confirmed in direct enzyme activity assay and further demonstrated indirectly by a thermal stabilization assay.

The increase in the thermal melting temperature of recombinant *Cs*CDPK1 in the presence of BKI 1369 indicates binding of either ligand to *Cs*CDPK1, based on previous studies showing correlations between binding affinities predicted by a thermal shift and direct measurement of enzyme inhibition by compounds (Ojo et al., 2016). Additionally, the IC_50_ concentration of BKI 1369 measured against recombinant *Cs*CDPK1 (4.5 nM) corroborated the efficacy of this compound in the *in vitro* dose-response assay (IC_50_ = 40 nM). Further validation of the relatedness of *Cs*CDPK1 to *Tg*CDPK1 was shown by improved EGTA thermal stabilization of the enzymes relative to the control where Ca^2+^ is presumably bound to the recombinant enzyme (Supplementary Fig. S2B) as previously reported (Scheele et al., 2018). This result seems counterintuitive since calcium activates the enzymes, yet chelating Ca^2+^ appears to maximize thermal stability (Scheele et al., 2018).

The *in vitro* IC_50_ and IC_95_ values obtained for BKI 1369 were in the nanomolar range, which supports data published for efficacy against *C. parvum* (Hulverson et al., 2017) and *C. hominis* (Lee et al., 2018). Treatment of *C. suis* infected IPEC-1 cells with IC_95_ concentrations of BKI 1369 for five days significantly reduced the number of free merozoites as seen in both light microscopy and TEM, without any apparent effect in the ultrastructure of *C. suis* merozoites. In *Toxoplasma* and *Neospora* treated with other BKIs such as BKI 1294, morphological alterations *in vitro* were only seen following longer treatment duration with a very high dose of 2.5 μM (Ojo et al., 2014; Winzer et al., 2015). However, this would not be an option in the *in vitro* model employed here, since the host cells are sensitive to higher concentrations of BKI 1369 and, more significantly, *C. suis* has a time-limited developmental phase terminating in oocyst production compared to the permanent lytic cycle of tachyzoite replication *in vitro* in *Toxoplasma* and *Neospora.*

BKIs have been shown to inhibit host cell invasion, egress and cytokinesis *in vitro,* as well as to reduce cerebral parasite burden in standardized murine models of cyst-forming apicomplexan parasites such as *T. gondii* and *N. caninum* (Doggett et al., 2014; Ojo et al., 2014; Winzer et al., 2015). In a calf clinical model and an acute porcine model of cryptosporidiosis, treatment with 10 mg/kg BW BKI 1369 twice daily for five days significantly reduced clinical diarrhea and fecal oocyst excretion and improved overall health in infected animals (Lee et al., 2018; Schaefer et al., 2016). The same treatment regime was adopted in the present *in vivo* study to evaluate the therapeutic efficacy of BKI 1369, a candidate BKI with fewer known side effects compared to other tested BKIs (Hulverson et al., 2017), against porcine cystoisosporosis in its natural host.

The experimental model of suckling piglet cystoisosporosis was previously employed to evaluate efficacy of anticoccidial treatment based on quantitative and qualitative oocyst shedding, fecal consistency/diarrhea, body weight development and general animal health (Joachim and Mundt, 2011; Joachim et al., 2014, 2018b; Mundt et al., 2006, 2007; Shrestha et al., 2015). In mammalian species an anticoccidial is considered to be efficient *in vivo* when oocyst shedding is noticeably reduced (indicating reduced environmental contamination with parasite stages, i.e. reduced infection pressure) and animal health and weight gain are improved compared to the mock-treated control (Joachim et al., 2018a). Treatment with BKI 1369 significantly reduced oocyst excretion and diarrhea in piglets infected with *C. suis* in the presence of other entero-pathogens. The high efficacy of BKI 1369 in reducing oocyst excretion and diarrhea is comparable to that of toltrazuril (Mundt et al., 2007; Joachim and Mundt, 2011; Joachim et al., 2018b) and was observed irrespective of the parasite strain employed, indicating that BKI 1369 therapy could be effective in combating toltrazuril susceptible as well as resistant *C. suis*.

It has been suggested that lesions induced by intracellular replication of *C. suis* impair physiological and functional balance of the intestine and therefore have a negative impact on body weight development of piglets (Mundt et al., 2006). A similar effect of *C. suis* infection was observed in this study with significantly lower daily body weight gain and total weight gain in the control piglets compared to BKI 1369 treated piglets during the acute phase of infection. This effect tended to wane in the following period (after SD 15), which might be due to gradual healing of the intestinal epithelium and maturation of the immune system with increasing piglet age, a general feature in coccidiosis. Histological sections from mock treated control piglets revealed cuboidal villous epithelium, shortened villi and villous fusion together with numerous merogonic stages of *C. suis* within parasitophorus vacuoles. In comparison, morphological alterations in the gut were not observed in treated piglets, which underpins the hypothesis that *C. suis-*induced intestinal damage disturbs the physiological function of the intestine, ultimately leading to diarrhea, malabsorption of nutrients and reduced weight gain (Lindsay et al., 1985; Stuart et al., 1982).

BKI 1369 achieved therapeutic concentrations in piglet plasma in the micromolar range, far above the effective *in vitro* levels (IC_95_ = 200 nM). The plasma concentration already reached a peak 2 h after the 1st dose and the concentration gradually increased to 11.7 μM 2 h before the 9th dose, indicating accumulation of the drug in plasma after treatment twice daily for 5 days. Consequently, assuming similar levels are found in infected gut epithelium, the *in vivo* parasites were constantly exposed to very high concentrations of BKI 1369. This could be the reason for the complete cessation of parasite development and replication *in vivo* compared to *in vitro* experiments. These results are in accordance with previous studies on BKI 1369 in *C. hominis* infected piglets, which reported low concentrations of the metabolites during the dosing period. Further pharmacokinetics studies in piglets to determine the concentrations of BKI 1369 in gastrointestinal tissues over time would be warranted to better understand the relationship between efficacy and exposure at the infection sites. Also, since the plasma levels reached the observed concentrations of 11.7 μM during dosing, well above the 1 μM concentration that showed effects *in vitro* to IPEC-1 cell viability, multi-dose toxicity studies that include blood chemistry and histopathology would be warranted to fully assess any effects that BKI 1369 might have on the host species. In summary, the present study evaluated therapeutic efficacy, pharmacokinetics and safety parameters of BKI 1369 against porcine cystoisosporosis in an experimental infection of its natural host. Application of single drug concentration is one of the major limitations of this study, due to which it was not possible to determine minimal effective drug concentration and to develop practical drug application scheme. Oral applications of BKI 1369 were highly effective in reducing oocyst shedding and diarrhea, and improved body weight gain in piglets infected with *C. suis*, including the toltrazuril-resistant Holland-I isolate, without adverse effects. The successful outcome in both the *in vitro* culture and the animal infection model of porcine cystoisosporosis also strongly argues for developing BKI 1369 as a chemotherapeutic compound against this disease, both in pigs and in other mammals including humans (Shrestha et al., 2015). However, BKI 1369 therapy in the field might have practicability issues, owing to the need for multiple doses. Therefore, future studies on BKI 1369 should be concentrated towards ease of drug handling and reducing treatment frequencies.

## Conflicts of interest

Dr. Wesley C. Van Voorhis is an officer and owns stock in ParaTheraTech Inc., a company that is trying to bring BKIs to the animal health market. He helped to design the experiments and edited the paper, but did not have a role in performing or interpreting the results.

## Appendix A Supplementary data

Supplementary Table S1 Overview of parasitological parameters for all experimental groups; MM: McMaster countable; AUC: area under the curve; OpG: oocysts per grams of feces; SD: standard deviation. For group sizes, see Table 1 Supplementary Table S1

Data Profile Data Profile
