# Supplementary material for: Bumped kinase inhibitor 1369 is effective against Cystoisospora suis in vivo and in vitro
Source: Int J Parasitol Drugs Drug Resist. 2019 Apr 2;10:9–19. doi: 10.1016/j.ijpddr.2019.03.004 (PMC6453670; doi:10.1016/j.ijpddr.2019.03.004)
Supplement: Supplementary Table S1 — Overview of parasitological parameters for all experimental groups; MM: McMaster countable; AUC: area under the curve; OpG: oocysts per grams of feces; SD: standard deviation. For group sizes, see Table 1 [file mmc1.docx]

| **Group** | **Total number of samples; 14 sampling days** | **% piglets with oocyst excretion** | **Mean excretion days ± SD** | **% days with MM excretion ± SD** | **Mean AUC for OpG;** | **Mean OpG ± SD** | **Max OpG; (study day)** |
| --- | --- | --- | --- | --- | --- | --- | --- |
| **Wien-BKI** | 140 | 0 | 0 | 0 | 0 | 0 | 0 |
| **Wien-Ctrl** | 126 | 100 | 5.3 ± 3.8 | 41.27 ± 24.7 | 122,914 | 17092 ±  21142.9 | 48063  (9) |
| **Holl-BKI** | 70 | 20 | 0.2 ± 0.4 | 1.43 ± 3.2 | 133 | 133 ±  297.8 | 666  (8) |
| **Holl-Ctrl** | 70 | 100 | 2.8 ± 1.3 | 21.43 ± 10.1 | 76,523 | 17898 ±  23929.1 | 65934  (12) |
